# 晚期非小细胞肺癌三线治疗疗效及生存分析

**DOI:** 10.3779/j.issn.1009-3419.2012.06.08

**Published:** 2012-06-20

**Authors:** 岚 邵, 正波 宋, 林 胡, 发君 谢, 广媛 娄, 卫 洪, 翠萍 古, 丹 洪, 宝钗 林, 沂平 张

**Affiliations:** 1 310053 杭州，浙江中医药大学第二临床医学院 Zhejiang Chinese Medical University, the Second Clinical Medical College, Hangzhou 310053, China; 2 310022 杭州，浙江省肿瘤医院化疗中心 Department of Medical Oncology, Zhejiang Cancer Hospital, Hangzhou 310022, China

**Keywords:** 肺肿瘤, 三线治疗, 化疗, 靶向治疗, 预后, Lung neoplasms, Tird-line treatment, Chemotherapy, Targeted therapy, Prognosis

## Abstract

**背景与目的:**

随着高效低毒药物的出现，越来越多的晚期非小细胞肺癌（non-small cell lung cancer, NSCLC）患者有机会接受三线治疗，但目前三线的标准治疗除厄洛替尼外尚无其它选择方案。本研究旨在比较单药化疗、靶向药物与双药联合化疗在晚期NSCLC患者三线治疗中的疗效与安全性。

**方法:**

回顾分析115例Ⅲb期/Ⅳ期接受三线治疗的NSCLC患者的疗效及生存状况。采用*Kaplan-Meier*曲线、*Cox*多因素生存分析模型进行单因素和多因素分析。

**结果:**

单药组、靶向治疗组与双药联合组中位无进展生存时间（progression free survival, PFS）分别为2.30个月、3.17个月和2.37个月（*P*=0.045），三线治疗后的中位生存时间（overall survival, OS）分别为8.00个月、10.40个月和7.87个月（*P*=0.110），Ⅲ度-Ⅳ度毒性反应发生率分别为33.3%、18.2%和68.8%（*P* < 0.001）。多因素分析显示体能状况（performance status, PS）评分（*P* < 0.001）是PFS的独立预后因素，既往无吸烟史（*P*=0.011）、PS评分0分-1分（*P* < 0.001）和一二线治疗疗效获得疾病控制（*P*=0.044）是三线治疗OS的独立预后因素。

**结论:**

PS评分较好、既往不吸烟和一二线治疗疗效疾病控制的患者在三线治疗中更能获益，与化疗单药或双药相比靶向药物组PFS显示出优势。

## Abstract (en)

**Background and objective:**

The appearance of highly effective and low toxic drugs enables an increasing number of advanced non-small cell lung cancer (NSCLC) patients to receive third-line therapy. No other standard choice for third-line therapy aside from erlotinib is possible. This study respectively explores the efficacy and safety of single chemotherapy, epidermal growth factor receptor-tyrosine kinase inhibitors (EGFR-TKIs), and doublet chemotherapy in advanced NSCLC third-line treatment.

**Methods:**

This study included 115 NSCLC patients in the stage Ⅲb or Ⅳ who were retrospectively reviewed to investigate the differences of survival time between different treatments. Univariate and multivariate analyses were conducted based on the *Kaplan-Meier* method and *Cox* proportional-hazards model.

**Results:**

The median progression free survival (PFS) values in the single agent, EGFR-TKIs and doublet groups were 2.30, 3.17 and 2.37 months, respectively (*P*=0.045). The median overall survival from the initiation of the third-line treatment were 8.00, 10.40 and 7.87 months in the three groups (*P*=0.110). The rates of stage Ⅲ-Ⅳ toxicities were 33.3%, 18.2% and 68.8% (*P* < 0.001), respectively. After the third-line treatment, the PFS was significantly increased in patients with a performance status (PS) of 0 to 1 (*P* < 0.001), and the survival time was prolonged in patients who never smoke (*P*=0.011), have good PS (*P* < 0.001), and have disease control after both first- and second-line treatments (*P*=0.044) using multivariate analysis.

**Conclusion:**

Advanced NSCLC patients who never smoke, have good PS scores, and have good disease control from the first- and second-line therapies could benefit more in third-line treatment. EGFR-TKIs therapy showed increased PFS compared with single and doublet agents.

肺癌是目前最常见的恶性肿瘤之一，其死亡率居恶性肿瘤之首^[1]^。肺癌中大约80%是非小细胞肺癌（non-small cell lung cancer, NSCLC）^[2]^。NSCLC预后较差，据美国2009年统计结果显示5年生存率仅为16%左右，且超过40%-50%的NSCLC患者在发现时已是晚期^[3]^。化疗给NSCLC患者带来了一定的生存获益，但因为化疗药物的毒性和易耐药性，患者往往会因毒副反应不能耐受而需要换药或疾病进展而需接受多线化疗。培美曲塞等新的低毒化疗药物的出现增加了晚期NSCLC患者接受进一步治疗的机会。研究^[4]^显示40%-60%的患者可以接受二线治疗，20%-30%的患者会接受三线治疗。已有多项研究^[5-7]^显示二线化疗会给患者带来生存获益和生活质量的改善，INTEREST^[8]^和BR21^[7]^临床试验也显示吉非替尼和厄洛替尼可以带来明显的生存获益。但是，目前除BR21研究纳入部分三线治疗患者外，还缺少大型前瞻性的研究和大样本回顾性的分析来研究三线治疗的意义^[9, 10]^。对于晚期NSCLC三线治疗的疗效及预后因素分析是值得关注的问题。基于此本研究组进行了一项回顾性研究，旨在探讨晚期NSCLC患者三线治疗疗效及与生存的关系。

## 1 资料与方法

### 1.1 临床资料

连续性收集2006年1月1日-2011年10月30日在浙江省肿瘤医院就诊并具有完整随访资料的Ⅲb期和Ⅳ期NSCLC患者，其中二线治疗失败后进入三线治疗的共134例，其中19例因治疗疗效不详而被排除，共115例患者纳入本研究。115例NSCLC患者在一线及二线治疗中接受靶向治疗、单药化疗和双药联合化疗分别为3例（2.6%）、10例（8.7%）和102例（88.7%）及42例（36.5%）、28例（24.3%）和45例（39.2%）。115例患者按三线不同治疗方案分成3组，包括靶向治疗组（epidermal growth factor receptor-tyrosine kinase inhibitors，EGFR-TKIs，表皮生长因子受体酪氨酸激酶抑制剂）44例（38.3%）、单药化疗组39例（33.9%）及双药联合化疗组32例（27.8%)。靶向治疗组：厄洛替尼20例，吉非替尼24例；单药化疗组：多西他赛19例，培美曲塞20例；双药联合化疗组：铂类联合培美曲塞15例，多西他赛13例，其它非铂联合4例。115例患者中仅不吸烟患者比例在3个治疗组之间的差异有统计学意义（*P*=0.045），其它临床特征的分布在三组之间无统计学差异。具体临床特征分布见表 1。

**1:** 115例接受三线治疗的Ⅲb期/Ⅳ期肺癌患者的临床特征 The clinical characteristics of 115 stage Ⅲb/Ⅳ lung cancer patients receiving third-line therapy

Variable	*n* (%)	EGFR-TKIs	Single-agent chemotherapy	Doublet chemotherapy	*P*
Gender					0.803
Male	67 (58.3%)	24 (54.5%)	24 (61.5%)	19 (59.4%)	
Female	48 (41.7%)	20 (45.5%)	15 (38.5%)	13 (40.6%)	
Performance status					0.232
0-1	78 (67.8%)	34 (77.3%)	24 (61.5%)	20 (62.5%)	
≥2	37 (32.2%)	10 (22.7%)	15 (38.5%)	12 (37.5%)	
Median age at diagnosis					0.402
≥65 year	29 (25.2%)	14 (31.8%)	9 (23.1%)	6 (18.8%)	
< 65 year	86 (74.8%)	30 (68.2%)	30 (76.9%)	26 (81.3%)	
Smoking status					0.045
No	68 (59.1%)	32 (72.7%)	18 (46.2%)	18 (56.3%)	
Yes	47 (40.9%)	12 (27.3%)	21 (53.8%)	14 (43.8%)	
Histology					0.683
Adenocarcinoma	92 (80.0%)	37 (84.1%)	30 (76.9%)	25 (78.1%)	
Non-adenocarcinoma	23 (20.0%)	7 (15.9%)	9 (23.1%)	7 (21.9%)	
Staging					0.401
Ⅳ	83 (72.2%)	30 (68.2%)	27 (69.2%)	26 (81.3%)	
Ⅲb	32 (27.8%)	14 (31.8%)	12 (30.8%)	6 (18.7%)	
Surgical history					0.638
No	96 (83.5%)	35 (79.5%)	34 (87.2%)	27 (84.4%)	
Yes	19 (16.5%)	9 (20.5%)	5 (12.8%)	5 (15.6%)	
EGFR-TKIs: epidermal growth factor receptor-tyrosine kinase inhibitors					

### 1.2 近期疗效评价

靶向治疗1个月后或化疗2个周期后评估近期疗效，对于疗效稳定或有效的患者，每2个月复查1次CT及其它影像学检查。根据实体瘤疗效评价标准（Response Evaluation Criteria in Solid Tumors, RECIST）1.1评价近期疗效，分为完全缓解（complete response, CR）、部分缓解（partial response, PR）、疾病稳定（stable disease, SD）和疾病进展（progressive disease, PD）。近期有效率（response rate, RR）＝（CR+PR）/（CR+PR+SD+PD）×100%。疾病控制率（disease control rate, DCR）＝（CR+PR+SD）/（CR+PR+SD+PD）×100%。一二线治疗疾病控制：一、二线治疗疗效均没有PD。一二线治疗进展：一、二线治疗任一疗效为PD。

### 1.3 随访和生存分析

随访采用门诊随访或电话方式，末次随访时间为2012年2月8日。三线治疗生存期（overall survival, OS）定义为患者自三线治疗开始至患者死亡或末次随访的时间。无进展生存期（progression-free survival, PFS）定义为患者自三线治疗开始至明确为PD的时间。

### 1.4 不良反应评价

根据美国国立癌症研究院通用毒性标准（common toxicity criteria, CTC）第3版评价不良反应（0-Ⅳ度）。

### 1.5 统计学分析

应用SPSS 17.0软件进行统计学分析。临床特征、疗效和毒性反应等比较采用卡方检验。PFS和OS采用*Kaplan-Meier*法及*Cox*模型进行预后分析。*P* < 0.05为差异有统计学意义。

## 2 结果

### 2.1 疗效反应

115例接受三线治疗的患者中，单药化疗组39例，靶向治疗组44例，双药联合化疗组32例。所有患者疗效均可评价，三组均无CR患者，单药化疗组、靶向治疗组和双药联合化疗组分别有1例（2.6%）、8例（18.2%）和3例（9.4%）达到PR，DCR分别为51.3%（20例），75.0%（25例）和40.6%（13例），三组PR及DCR差异均无统计学意义（*P*>0.05）。三组中位PFS分别为2.30个月、3.17个月和2.37个月，差异有统计学意义（*P*=0.045，图 1A）；三组中位OS分别为8.00个月、10.40个月和7.87个月，差异无统计学意义（*P*=0.110，图 1B）。将单药与双药合并为化疗组，与EGFR-TKIs靶向治疗组对比发现，中位PFS分别为2.37个月和4.00个月（*P*=0.014，图 1C），中位OS分别为8.00个月和10.40个月（*P*=0.048，图 1D）。

**1:**
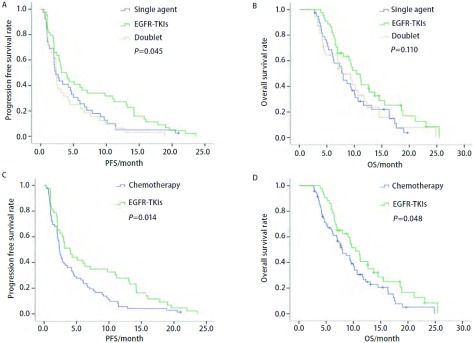
115例三线治疗Ⅲb期/Ⅳ期肺癌患者的三线PFS/OS对比曲线。A：三组的PFS曲线；B：三组的OS曲线；C：靶向治疗组与化疗组的PFS曲线；D：靶向治疗组与化疗组的OS曲线。 The third-line PFS/OS curves of 115 stage Ⅲb/Ⅳ lung cancer patients receiving third-line therapy. A: PFS curves of patients receiving third-line therapy between three groups; B: OS curves of patients receiving third-line therapy between three groups; C: PFS curves of patients receiving third-line therapy between EGFR-TKIs and chemothearpy; D: OS curves of patients receiving third-line therapy between EGFR-TKIs and chemothearpy. PFS: progression free survival; OS: overall survival.

### 2.2 毒性反应

115例三线治疗患者均可进行毒副反应评估。6例患者因严重的毒性反应拒绝进一步治疗（1例感染，3例贫血，2例白细胞减少），4例患者从双药联合化疗组换至单药化疗组。Ⅲ度/Ⅳ度毒性反应总发生率为37.4%，其中EGFR-TKIs组为18.2%（8/44），单药化疗组为33.3%（13/39），双药联合化疗组为68.8%（22/32），三组间差异有统计学意义（*P* < 0.001）。具体见表 2。

**2:** 115例三线治疗Ⅲb期/Ⅳ期肺癌患者的Ⅲ度-Ⅳ度毒性反应 The toxic side effects of 115 stage Ⅲb/Ⅳ lung cancer patients receiving third-line therapy

Toxic side effects	EGFR-TKIs (*n*=44)			Single agent (*n*=39)			Doublet agent (*n*=32)		*P*
	Ⅲ	Ⅳ		Ⅲ	Ⅳ		Ⅲ	Ⅳ	
Hematologic	0	0		6	1		9	6	< 0.001
Digestive	2	0		3	0		2	0	0.835
Diarrhea	2	0		1	0		2	0	0.241
Hepatic and renal	0	0		1	0		1	0	0.524
Fatigue	0	0		1	0		1	0	0.337
Rash	3	1		0	0		0	0	0.570
Fever with neutrophils	0	0		0	0		0	1	0.270

### 2.3 预后因素分析

多因素分析结果显示，三线治疗前体能状况（performance status, PS）评分（HR=2.61, *P* < 0.001）是三线PFS的独立影响因素，PS评分在0分-1分的PFS明显优于2分的患者。三线治疗前PS评分（HR=3.11, *P* < 0.001）、吸烟史（HR=1.86, *P*=0.011）和一二线治疗疗效（HR=1.61, *P*=0.044）是三线治疗OS的独立影响因素，PS评分0分-1分、既往不吸烟、一二线治疗疾病控制的患者预后较好。见表 3。

**3:** 115例三线治疗的Ⅲb期/Ⅳ期肺癌患者三线PFS和OS多因素分析 Multivariate analysis of PFS and OS (from the initiation of third-line treatment) for 115 stage Ⅲb/Ⅳ lung cancer patients receiving third-line therapy

Variable	PFS			OS	
	HR（95%CI）	*P*		HR（95%CI）	*P*
Gender	0.77 (0.50-1.17)	0.222		0.79 (0.50-1.26)	0.324
Age	0.84 (0.53-1.31)	0.441		1.52 (0.91-2.53)	0.107
Stage	0.79 (0.50-1.25)	0.313		0.64 (0.37-1.10)	0.108
Surgical history	0.84 (0.48-1.47)	0.541		1.01 (0.54-1.88)	0.982
Smoking history	1.38 (0.90-2.11)	0.146		1.86 (1.16-2.99)	0.011
Histology	0.86 (0.51-1.47)	0.589		0.91 (0.48-1.70)	0.758
Performance score	2.61 (1.62-4.20)	< 0.001		3.11 (1.91-5.06)	< 0.001
Response to prior treatments	0.74 (0.49-1.11)	0.142		1.61 (1.01-2.57)	0.044
Third-line drug	1.07 (0.83-1.38)	0.593		1.01 (0.76-1.35)	0.948

进一步分析一二线治疗疗效对三线治疗的影响，一二线治疗疾病控制和进展的中位OS分别为11.20个月和6.67个月，具有统计学差异（*P*=0.005，图 2A）。在一二线治疗中有45例曾接受过靶向治疗（一线3例，二线42例），分析发现既往靶向治疗亚组疗效为疾病控制和进展的三线治疗PFS分别为2.43个月和2.40个月，差异无统计学意义（*P*=0.528，图 2B）。

**2:**
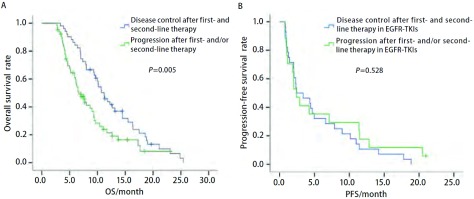
115例三线治疗Ⅲb期/Ⅳ期肺癌患者一二线治疗疾病控制和进展的三线OS/PFS的对比曲线 The third-line OS(A) and PFS(B) curves of 115 stage Ⅲb/Ⅳ lung cancer patients received third-line therapy by response disease control and progression after first- and/or second-line therapy

## 3 讨论

随着新的高效而低毒的化疗和靶向药物的出现，患者在接受标准一、二线治疗后越来越多的患者仍然有机会接受三线及以上治疗。目前，EGFR-TKI在晚期NSCLC治疗中已被证实有较好的临床获益，在临床上也常用于二、三线治疗^[7, 8]^。2012年NCCN指南中晚期NSCLC的三线治疗仅推荐厄洛替尼，指出其优于最佳支持治疗。在中国未使用过EGFR-TKIs的患者还可以选择吉非替尼^[11]^。到目前为止，晚期NSCLC三线及以上治疗方案中还没有研究数据显示细胞毒类化疗药物与靶向药物在患者生存上的优劣对比。

本研究纳入115例三线治疗患者，分析结果显示三线治疗前PS评分（*P* < 0.001）、吸烟史（*P*=0.011）和一二线治疗疗效（*P*=0.044）是生存的独立预后因素，PS评分2分、有吸烟史及一二线治疗进展的患者预后较差。Girard等^[10]^回顾性分析了613例NSCLC化疗患者，其中173例接受了三线治疗（细胞毒药物化疗131例，表皮生长因子受体酪氨酸激酶抑制剂42例），研究结果提示接受三线治疗的最佳获益人群是PS评分0分-1分（HR=0.81, 95%CI: 0.76-0.86, *P*=0.008）和一二线治疗获得疾病控制的患者（HR=0.47, 95%CI:0.33-0.67, *P*=0.001）。Scartozzi等^[12]^的回顾性研究也显示二线治疗疗效是三线治疗患者OS的影响因素（*P*=0.03）。这些结论也与我们的研究结果相符，PS评分较好的患者往往会接受多线的治疗，本研究中PS 0分-1分的患者占67.8%也可能是影响预后的原因。本项研究中一二线治疗疗效对三线PFS和OS影响明显，是独立预后因素，这与Girard等^[10]^和Scartozzi等^[12]^的研究结果也符合，提示既往化疗或靶向治疗有效的患者进展后应该积极接受后续的治疗。对既往一二线治疗中曾接受过靶向治疗的45例患者（一线3例，二线42例）分析显示，既往靶向治疗亚组疗效获疾病控制和进展与三线治疗PFS之间无关（*P*=0.528），表明既往化疗的疗效可能更能影响三线治疗的疗效，而靶向治疗影响不大，其原因有待于进一步探讨。

本研究中吸烟是影响预后的独立因素（*P*=0.011），从不吸烟的患者较吸烟患者预后好。单药组不吸烟的患者比例高于其余两组（*P*=0.045）。进一步的分析结果显示单药组、靶向治疗组和双药联合组不吸烟与吸烟患者的中位OS分别为10.67个月*vs* 7.33个月，11.07个月*vs* 9.43个月，10.17个月*vs* 7.03个月，两两比较后均无统计学差异（*P*>0.05）。因此，吸烟比例不同不足以对三组的OS产生明显影响。

目前NSCLC二线及以上治疗的临床实践原则多为单药治疗，因为单药的毒性相对小，患者在接受多线治疗后耐受相对较差。但2009年的一项*meta*分析^[13]^显示在NSCLC二线治疗中，联合铂类治疗与单药治疗的客观有效率（objective response rate, ORR）分别为15.1%和7.3%（*P*=0.000, 4），中位PFS分别为14周和11.7周（*P*=0.000, 9），但两组的OS无差异（*P*=0.32）。在毒性方面，Ⅲ度-Ⅳ度血液学毒性和非血液学毒性双药较单药明显（41% *vs* 25%, *P* < 0.000, 1）。虽然二线治疗双药与单药治疗相比并未延长OS且毒副作用较大，但在ORR及PFS上仍有优势。Chen等^[14]^的研究发现双药化疗也是一种可行的方案，特别是在PS评分较好、一线或二线使用靶向药物的情况下。在其它一部分研究^[15, 16]^中联合方案相对单药而言OS也有一定的提高。目前，NSCLC患者能否在三线及以上治疗中获得可持续的PFS仍然没有得到定论。考虑到顺铂和卡铂的毒性和交叉耐药，本研究三线联合化疗方案中大多数使用的是奥沙利铂或奈达铂。结果显示联合化疗方案相对单药方案的PFS和OS略好，但明显不及靶向治疗，且毒性明显增加。单因素分析三种方案对中位PFS的影响有统计学意义（*P*=0.045），将化疗单药和双药组合并后与靶向组对比的单因素分析显示，靶向组在PFS（*P*=0.014）和OS（*P*=0.048）方面均要优于化疗组。三线治疗Ⅲ度-Ⅳ度毒性反应发生率为37.4%，其中靶向治疗组为18.2%（8/44），单药化疗组为33.3%（13/39），双药联合化疗组为68.8%（22/32），提示靶向治疗在三线治疗中是可以优先考虑的，这也与BR21研究^[7]^证实厄洛替尼在三线治疗中可以为患者带来生存获益的结果符合。

综上，临床上选择晚期NSCLC患者接受三线治疗时可根据临床特征如PS评分、吸烟史、一二线治疗的疗效等，在治疗方案的选择上可考虑靶向药物或单药化疗。由于本研究为回顾性分析，样本量相对偏小，结果有待进一步大样本和前瞻性研究证实。
